# Insights into Recent Studies on Biotransformation and Pharmacological Activities of Ginsenoside Rd

**DOI:** 10.3390/biom12040512

**Published:** 2022-03-28

**Authors:** Xiaoping Song, Lina Wang, Daidi Fan

**Affiliations:** 1 Shaanxi Key Laboratory of Degradable Biomedical Materials, School of Chemical Engineering, Northwest University, 229 Taibai North Road, Xi’an 710069, China; 2 Shaanxi R&D Center of Biomaterials and Fermentation Engineering, School of Chemical Engineering, Northwest University, 229 Taibai North Road, Xi’an 710069, China; 3 Biotechnology & Biomedicine Research Institute, Northwest University, 229 Taibai North Road, Xi’an 710069, China; wanglina1@stumail.nwu.edu.cn

**Keywords:** ginsenoside Rd, biotransformation, pharmacological activities

## Abstract

It is well known that ginsenosides—major bioactive constituents of Panax ginseng—are attracting more attention due to their beneficial pharmacological activities. Ginsenoside Rd, belonging to protopanaxadiol (PPD)-type ginsenosides, exhibits diverse and powerful pharmacological activities. In recent decades, nearly 300 studies on the pharmacological activities of Rd—as a potential treatment for a variety of diseases—have been published. However, no specific, comprehensive reviews have been documented to date. The present review not only summarizes the in vitro and in vivo studies on the health benefits of Rd, including anti-cancer, anti-diabetic, anti-inflammatory, neuroprotective, cardioprotective, ischemic stroke, immunoregulation, and other pharmacological effects, it also delves into the inclusion of potential molecular mechanisms, providing an overview of future prospects for the use of Rd in the treatment of chronic metabolic diseases and neurodegenerative disorders. Although biotransformation, pharmacokinetics, and clinical studies of Rd have also been reviewed, clinical trial data of Rd are limited; the only data available are for its treatment of acute ischemic stroke. Therefore, clinical evidence of Rd should be considered in future studies.

## 1. Introduction

Ginseng (Panax ginseng C.A. Mey, a perennial herb of the Araliaceae family) is conventionally used as a tonic herbal medicine and a functional food, it is receiving more attention due to its remarkable beneficial pharmacological activities. Ginsenosides are major bioactive constituents of ginseng, of which, nearly 150 have been isolated and identified from roots, fruits, leaves, and flower buds of ginseng [1]. Ginsenosides directly extracted from Araliaceae plants (Panax ginseng, Panax quinquefolium, Panax notoginseng, etc.) are called naturally prototype ginsenosides, also known as main ginsenosides due to their relatively high contents, mainly including Ra, Rb1, Rb2, Rb3, Rc, Rd, Re, Rf, Rg1, etc. Rare ginsenosides or minor ginsenosides are the metabolites of prototype ginsenosides catalyzed by enzymes, including Rh2, Rg3, Rk2, Rh3, Rk1, Rg5, Rk3, Rh1, Rh3, Rh4, CK, etc., which have much higher anti-cancer activities and are more easily absorbed by the human body [2].

Ginsenoside Rd, belonging to protopanaxadiol (PPD)-type ginsenosides, exhibits diverse and powerful pharmacological activities, including anti-inflammatory, anti-tumor, neuroprotective effects, cardiovascular protection, immunoregulation, and other beneficial health effects. However, the content of ginsenoside Rd in wild ginseng is very low, and traditional chemical conversion production methods, such as heating, mild acid hydrolysis, and alkali treatment, display some unavoidable disadvantages, such as a lower yield and more side reactions due to non-specific reactions. Therefore, studies have been conducted on the biotransformation of ginsenosides Rb1, Rb2, and Rc, due to the advantages of biotransformation (e.g., high selectivity, environmentally-friendly, etc.). Ginsenoside Rd, in view of its high levels of safety and diverse biological functions, may be a potential therapeutic agent for many diseases, in particular, neurological diseases, cardiovascular diseases, and metabolic diseases. 

Researchers, in previous studies, have delved into the promising role of ginsenoside Rd on ischemic stroke and its neuroprotective effects [3,4]. The present paper, however, differs significantly from previous works, not only by including detailed information on the biotransformation of Rd, but also by including clinical pharmacokinetic studies, exploring the anti-cancer, anti-inflammatory, antioxidative, neuroprotective, cardiovascular protection, and immunoregulation effects, as well as other in vitro and in vivo pharmacological activities.

## 2. Biotransformation

**Figure 1:**
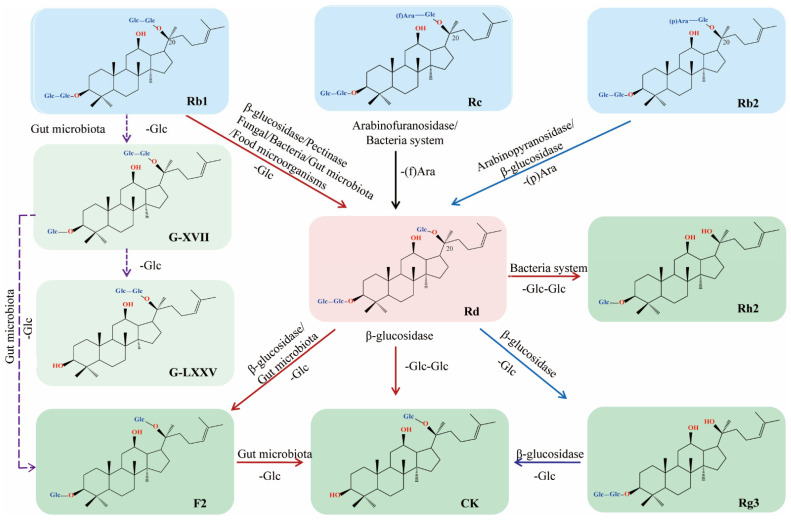
Schematic illustration of biotransformation of major ginsenosides Rb1, Rb2, and Rc to Rd (⟶ major pathway; ⤏ minor pathway).

The content of ginsenoside Rd differs from different wild ginseng and parts of ginseng, and ranges from 0.02% to 1.66% [4]. Moreover, it is difficult and costly to isolate Rd from natural products; thus, a microbial enzymatic transformation has become the predominant conversion modality of ginsenoside Rd due to its distinct selectivity, mild reactive conditions, and environmental compatibility (Figure 1).

### 2.1. Enzymatic Transformation

Ginsenoside Rd—characterized by tetracyclic, dammarane-type triterpenes with three sugar moieties—is structurally similar to Rb1, Rb2, and Rc, but lacks one outer glycoside moiety at the C-20 position. The preparation of ginsenosides via hydrolysis of glycosidic bonds using enzymatic transformation methods has exceptional advantages, e.g., high selectivity, mild reactive conditions, and being environmentally-friendly. Therefore, it is viable to obtain ginsenoside Rd from Rb1, Rb2, and Rc by hydrolyzing the monosaccharide residue using a specific glycosidase, such as α-l-arabinofuranosidase, α-l-arabinopyranosidase, β-glucosidase, or pectinase (Table 1).

#### 2.1.1. Arabinofuranosidase

Ginsenoside Rc, one of the major components of ginseng, comprising 7–22% of total ginsenoside, has an arabinofuranosyl moiety and three glucopyranosyl moieties; thus, arabinofuranosidase and glucosidase could convert Rc to a deglycosylated ginsenoside [5]. In order to improve the biotransformation rate of ginsenoside Rd and optimize the enzymatic properties, studies about arabinofuranosidase have emerged in recent years. An et al. [5] identified a recombinant α-l-arabinofuranosidase AbfA, which was cloned from a soil bacterium; *Rhodanobacter ginsenosidimutans* Gsoil 3054T could biotransform ginsenoside Rc to ginsenoside Rd. Recombinant AbfA demonstrated substrate-specific activity for the bioconversion of ginsenosides, as it only hydrolyzed arabinofuranoside moieties from ginsenoside Rc and derivatives, not other sugar groups from ginsenosides Rb1 or Rb2. The following year, the same research team reported a novel recombinant α-l-arabinofuranosidase (Abf22-3) from the ginsenoside converting *Leuconostoc* sp. 22-3 isolated from the Korean fermented food kimchi, which could biotransform ginsenoside Rc into Rd [6]. Results showed that over 99.5% of Rc was converted to Rd after 24 h under optimal conditions of pH 6.0 and 30 °C. In another study, a molar yield of ginsenoside Rd was nearly 100% using a thermostable recombinant α-l-arabinofuranosidase from *Caldicellulosiruptor saccharolyticus* at a pH 5.5 and at 80 °C [7]. Later, Xie et al. [8] cloned and overexpressed the novel thermostable α-l-arabinofuranosidase (Tt-Afs) from *T. thermarum* DSM5069, which showed a high conversion and productivity of Rd. In addition, the ginsenoside Rc-hydrolyzing α-l-arabinofuranosidase gene, BsAbfA, was cloned from *Bacillus subtilis* and optimized. The results of molecular docking and site-directed mutagenesis suggested that the E173 and E292 variants for BsAbfA were important in effectively recognizing ginsenoside Rc, providing an effective biotransformation pathway of ginsenoside Rc into Rd [9].

Among the major ginsenosides, ginsenoside Rb2 accounts for 1–22% of the total ginsenosides in ginseng root [32] and could also be used for converting into Rd. Kim et al. [10] reported a recombinant enzyme α-l-arabinopyranosidase (AbpBs), which could efficiently catalyze the conversion of ginsenoside Rb2 to Rd by selectively hydrolyzing the outer arabinopyranoside moiety at the C-20 position.

#### 2.1.2. β-glucosidase

β-glucosidases, a heterogeneous group of enzymes, are capable of cleaving the β-glycosidic linkages of aryl and alkyl β-glucosides, β-linked oligoglucosides, and several other oligosaccharides. Some recombinant enzymes, especially β-glucosidases with different substrate specificities, have been widely applied to produce the rare ginsenosides. To date, considerable attention has been placed on the transformation of ginsenoside Rb1 into Rd with the use of β-glucosidases. The thermostable β-glucosidase Tt-BGL from extremophile *Thermotoga thermarum* DSM5069 selectively converts ginsenoside Rb1 into ginsenoside Rd, with high productivity [11]. Additionally, ginsenoside Rd has been used as an intermediate for the transformation of other rare ginsenosides. Quan et al. [12] reported that the recombinant β-glucosidase Bgp3 from *Microbacterium esteraromaticum* isolated from the ginseng field could catalyze the conversion of ginsenoside Rb1 to the more pharmacologically active major ginsenoside Rd and ginsenoside CK. Subsequently, they isolated a novel recombinant glycosidase Bgp2 from *Microbacterium esteraromaticum*, which belonged to the glycosyl hydrolase family 2 protein and could hydrolyze the ginsenoside Rb2 along the following pathway: Rb2 ⤏ Rd ⤏ 20(S)-Rg3 through the selective hydrolysis of the arabinopyranose and glucose moieties [13]. The ginsenoside-hydrolyzing β-glucosidase gene Bgy2, a member of the glycosyl hydrolase family 3 protein, was cloned and identified from *Lactobacillus brevis* [14]. Under the optimal conditions (pH 7.0, 30 °C), 1.0 mg/mL ginsenoside Rb1 was converted into 0.59 mg/mL ginsenoside Rd, with molar conversion productivities of 69%. Moreover, Rb1-hydrolyzing β-glucosidase from *Aspergillus niger* KCCM 11,239 was studied (and optimized) by Chang et al. [15]. The enzyme hydrolyzed β-(1⤏6)-glucoside at the C-20 position of ginsenoside Rb1 to generate Rd and Rg3, and hydrolyzed β-(1⤏2)-glucoside at the C-3 position to generate F2.

#### 2.1.3. Pectinase

Pectinase specifically hydrolyzes protopanaxadiol (PPD)-type ginsenosides and is a selective enzyme that converts ginsenoside Rb1 to Rd. Fang et al. [16] explored one-pot production process of ginsenoside Rd by coupling enzyme-assisted extraction with selective enzymolysis, and provided a higher yield at 52.5 °C and pH 6.0, suggesting that pectinase could be used as an efficient enzyme for producing ginsenoside Rd.

### 2.2. Microbial Transformation

Microbial transformation is also a major production method of Rd. The mechanism of enzymatic transformation involves hydrolyzing ginsenosides using the catalytic activity of the enzyme, which has the advantages of a short reaction cycle, low pollution, and high product purity; however, the reaction conditions are difficult to control, the enzyme is easy to inactivate, and the separation and purification processes of the enzymes are complicated. In contrast, microbial transformation is characterized by low costs, few byproducts, and wide applications, but the drawbacks of a long conversion time and a low biotransformation rate are inevitable. Therefore, the enzymatic–microbial transformation of ginsenosides have their own characteristics and complement each other in the actual production process. The production of Rd could be achieved through microbial methods, including fungus, bacteria, gut microbiota, and food microorganisms (Table 1).

#### 2.2.1. Fungal System

A mutant filamentous fungus *Paecilomyces bainier* 229-7 that transformed ginsenoside Rb1 to Rd with high selectivity and substrate tolerance was obtained (and identified) by Feng et al. [17]. The highly substrate-tolerant mutant produced ginsenoside Rd from Rb1 with a bioconversion rate as high as 94.9% under optimized culture conditions in shake flasks, along with an 89% bioconversion rate in 10 L fermenter, with a chromatographic purity of 92.6% purified by macroporous resin, which rendered it a promising strain for the preparation of Rd in the pharmaceutical industry. Later, the same team reported on the effects of external calcium treatments on the biotransformation of ginsenoside Rb1 to ginsenoside Rd by *Paecilomyces bainier* 229-7. Results suggested that both Ca^2+^ channels and calmodulin (CaM) were involved in ginsenoside Rd biotransformation via regulation of β-glucosidase activity [18]. Additionally, ginsenoside Rb1-converting fungus *Aspergillus versicolor* LFJ1403 was isolated and identified from the ginseng field soil and the biotransformation of ginsenoside Rb1 to Rd using an extracellular enzyme directly from the fungus spore production phase was investigated. The results of HPLC showed that Rd was the only product in this process, and the conversion rate was increased to 96% in shake flasks, indicating that the spore suspension biotransformation system had potential in the industrial production of Rd [19]. A novel ginsenoside Rd transformation fungus, *Aspergillus niger* TH-10a obtained from screening the survival library of LiCl and UV irradiation, could efficiently convert ginsenoside Rd from Rb1, and achieve the highest transformation rate of about 86% at 32 °C and pH 5.0 [20].

#### 2.2.2. Bacteria System

Some studies have focused on the discovery and identification of ginsenoside-transforming bacteria. To identify a microorganism that was capable of converting Rb1 into other ginsenosides, 12 *Microbacterium* spp. were screened by Hansoo et al. [21], and *M. trichothecenolyticum* was identified to convert Rb1 into Rd and then into Rh2 based on TLC and HPLC analyses of reaction products. Then, Akter et al. [22] isolated a gram-positive, aerobic, motile, rod-shaped bacterial strain (MAH-16T) from a soil sample of a vegetable garden and identified it as a member of the genus *Paenibacillus barengoltzii* SAFN-016T according to the 16S rRNA gene sequence comparisons, which might be responsible for the biosynthesis of ginsenoside Rd from major ginsenoside Rb1. Later, they also isolated a novel, gram-positive, and ginsenoside-converting bacterium (MAHUQ-46T) from forest soil, which was closely related to *Paenibacillus pinihumi* S23T (97.3% similarity) [23]. Furthermore, a Gram-negative, strictly aerobic, non-spore-forming, and rod-shaped bacterial strain (FW-6T) was isolated from a freshwater sample and displayed β-glucosidase activity that could transform ginsenoside Rb1 to Rd [24].

There are various microorganisms in the ecological environment of plants; some are attached to the surfaces of plants, while others live in the plants. Endophytes are fungi or bacteria commonly found in higher plants that live in the tissues and organs of healthy plants for some (or all) of their stages. Previous research focused on microorganisms that attached to plant surfaces and the rhizosphere, but the study of endophytes in plants was fledgling. An endophytic bacterium, G9y, with the ability to specifically convert ginsenoside Rc to Rd, was isolated from Panax quinquefolius; the transformation mechanism might be related to the production of α-l-arabinofuranosidase, which specifically hydrolyzes the terminal arabinofuranosyl moieties at the C-20 position of ginsenoside Rc [25]. Ginsenoside Rc was completely converted to Rd by bacterium G9y within 25 h after inoculation under the optimized conditions of pH 7.0 and 45 °C.

#### 2.2.3. Gut Microbiota and Food Microorganisms

Gut microbiota mainly function in the biotransformation of prototype ginsenosides into rare bioactive metabolites. When incubated anaerobically with pooled gut bacteria, including human gut bacteria [26], *Leuconostoc mesenteroides* DC102 [27], *Lactobacillus paralimentarius* [28], and probiotics [29], Rb1 generated five metabolites, namely Rd, F2, CK, and the rare gypenosides XVII (G-XVII) and LXXV (G-LXXV). Biocatalytic methods using probiotic enzymes for producing deglycosylated ginsenosides, such as Rd, have a (growing) role in the functional food industry. *Lactobacillus rhamnosus* GG, one of the most well-known probiotic bacteria, could be successfully used to convert ginsenoside Rb1 into Rd at the pH 6.0 and 40 °C [30]. *Dekkera anomala* YAE-1 strain separated from “airag” (Mongolian fermented mare’s milk) could produce β-glucosidase and has shown great capacity in converting ginsenoside Rb1 to Rd at 40 °C, pH 5.0 [31].

## 3. Pharmacological Activity

Ginsenoside Rd is known for its beneficial pharmacological activities. To date, extensive studies of in vitro cell biology and in vivo animal models have demonstrated that ginsenoside Rd offers potential anti-cancer, anti-diabetic, anti-inflammatory, neuroprotective, cardioprotective, ischemic stroke, immunological, and other pharmacological activities. In this section, we summarize recent studies on various health-promoting activities of ginsenoside Rd to provide a systematic summary and analysis of the pharmacological effects and the potential molecular mechanisms.

### 3.1. Anti-Cancer

**Figure 2:**
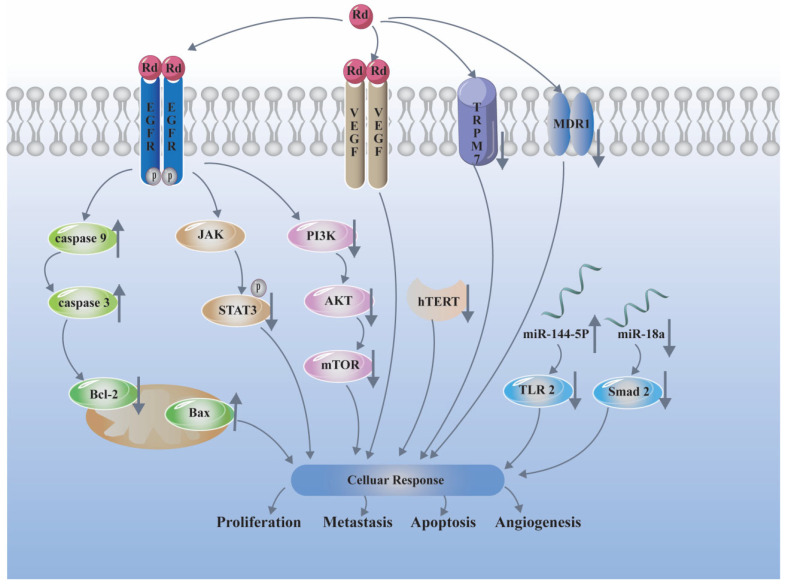
Anti-cancer mechanism of ginsenoside Rd. “↓” means downregulation, “↑” means upregulation.

The promising anti-cancer activity of ginsenoside Rd has been identified in various types of cell lines and animal models, including gastric cancer, colorectal cancer, lung cancer, breast cancer, glioblastoma, etc. (Table 2). The underlying anti-cancer mechanisms of ginsenoside Rd are shown in Figure 2. Ginsenoside Rd significantly inhibits cell proliferation and induces cell cycle arrest and cell apoptosis by increasing the expression of caspase-3, caspase-9, and the ratio of Bax/Bcl-2 in human gastric cancer [33], cervical cancer Hela cells [34], and human glioma U251 cells [35]. The possible mechanisms of Rd inhibiting glioma cells might be related to inhibition of telomerase activity by downregulating human telomerase catalytic subunit (hTERT) expressions at both mRNA and protein levels [35]. Moreover, it was reported that Rd reduced the proliferation and migration of glioblastoma cells by upregulating the tumor suppressor miR-144-5p and downregulating its target toll-like receptor 2 [36]. Lee et al. [37] identified fourteen proteins contributing to cell growth inhibition after ginsenoside Rd treatment in HT29 through two-dimensional gel electrophoreses, MALDI-TOF and TOF-MS, including proteins associated with mitosis (such as stathmin 1, microtubule-associated protein RP/EB family, stratifin) and associated with apoptosis (Rho GDP dissociation inhibitor, tropomyosin 1, annexin 5). The combination of combretastatin A4 phosphate (CA4P), a vascular disrupting agent, and Rd, had synergistic anti-tumor effects in hepatocellular carcinoma, which the mechanism might be related to the inhibition of HIF-1α via PI3K/AKT/mTOR signaling pathway [38]. The divalent cation–selective channel transient receptor potential melastatin 7 (TRPM7) channel was shown to affect the proliferation of some types of cancer cells. Several studies reported that ginsenoside Rd inhibited the proliferation and survival of gastric and breast cancer cells by inhibiting TRPM7 channel activity [39,40]. Moreover, ginsenoside Rd significantly inhibited metastasis in the human hepatocellular carcinoma, colorectal cancer, and breast cancer [41,42,43]. Research by Wang et al. [43] showed that Rd treatment attenuated breast cancer metastasis in part through derepressing miR-18a-mediated Smad2 expression regulation. A blockade of angiogenesis was an important approach for cancer treatment and prevention; thus, some studies investigated the effects of ginsenoside Rd on angiogenesis, in vitro and in vivo. Results demonstrated that Rd inhibited VEGF-induced migration, tube formation, and proliferation of primary cultured human umbilical vascular endothelial cells (HUVECs) dose-dependently [44]. Furthermore, Rd normalized the structure of tumor vessels, and improved the anti-tumor effect of 5- FU in xenograft mice [45]. Clinical drug resistance to chemotherapy is always considered a major obstacle in the successful treatment of cancer. Notably, ginsenoside Rd was reported to reverse doxorubicin resistance in MCF-7/ADR cells through downregulating the multidrug resistance 1 (MDR1) protein [46]. In addition, Rd could overcome cisplatin resistance in NSCLC by downregulating the nuclear factor erythroid 2-related factor 2 (NRF2) pathway [47].

Manipulation of gut microbiota composition through the treatment of prebiotics could be a novel preventive measure against cancer development. Interestingly, Rd exerted anti-cancer effects by holistically reinstating mucosal architecture, improving mucosal immunity, promoting beneficial bacteria, and downregulating cancer–cachexia associated bacteria [48]. 

### 3.2. Anti-Diabetic

Diabetes mellitus is characterized by chronic hyperglycemia, which also results in the abnormal accumulation of methylglyoxal (MG, one of the most reactive advanced glycation end-product precursors) and induces neuronal cell death in the central nervous system. Ginsenoside Rd and Rh2 were shown to ameliorate the cell viability of MG-treated astrocytes and improve insulin signaling, indicating that Rd and Rh2 might have therapeutic potential in treating diabetes-induced neurodegeneration [49]. Kaviani et al. [50] evaluated the effects of ginsenoside Rd on the apoptosis-associated cell death in human pancreatic islets, and results showed that Rd inhibited the progress of death of cultured human pancreatic islets by diminishing the apoptosis of the islet cells. Moreover, Jung et al. [51] developed a new pectin lyase-modified ginseng (GS-E3D), with enhanced ginsenoside Rd content, which had a potent protective role in diabetes-induced renal dysfunction through antioxidative and antiapoptotic activities. Diabetic retinopathy (DR) is a complex complication of diabetes that can lead to blindness. A recent report demonstrated that ginsenoside Rd ameliorated diabetes-driven vascular damage through enhancement of AMPK/SIRT1 interaction, which supported the potential vascular protective evidence of Rd for early DR [52] (Table 2).

### 3.3. Anti-Inflammatory and Antioxidative

**Figure 3:**
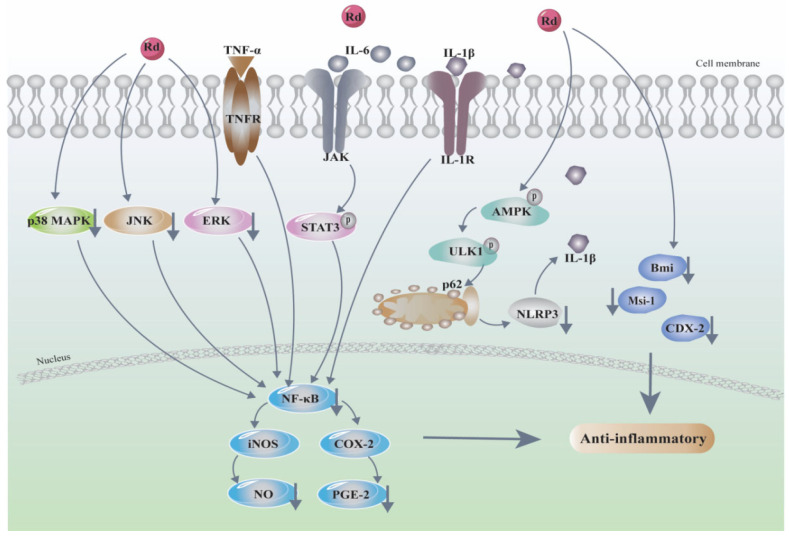
Anti-inflammatory mechanism of ginsenoside Rd. “↓” means downregulation.

Inflammatory response is a complex network composed of multiple mediators, cells, and pathways, which is involved in the occurrence and development of various diseases, such as cancer, atherosclerosis, and neurodegenerative diseases. Ginsenoside Rd exhibited significant anti-inflammatory activities against many inflammatory diseases, such as chronic hepatitis [53], neuroinflammation [54], osteoarthritis [55], and gastritis [56], through the downregulation of inducible nitric-oxide synthase (iNOS) and COX-2 by inhibiting NF-κB, furthering the inhibition of the production of NO and PGE2 [57,58] (Table 3). However, recent studies have strengthened the understanding of the mechanistic implications at molecular and cellular levels. The anti-inflammatory mechanism of ginsenoside Rd is shown in Figure 3. 

Ginsenoside Rd ameliorated colitis by inducing p62-driven mitophagy-mediated NLRP3 inflammasome inactivation and upregulating of AMPK/ULK1 signaling pathway in DSS-induced murine colitis model [59]. Moreover, ginsenoside Rd attenuated the inflammatory response in rats with TNBS-induced relapsing colitis and recurrent ulcerative colitis via modulating p38 and JNK signaling pathways, inhibiting neutrophil infiltration and promoting the antioxidant capacity [60,61]. A recent study evaluated the utility of Rd in gastrointestinal mucosal regeneration and clarified that Rd could stimulate the proliferation and differentiation of endogenous intestinal stem cells and improve recovery of intestinal function in a rat model of inflammatory bowel disease (IBD) by increasing the expression levels of Bmi, CDX-2, and Msi-1 [62]. In addition, ginsenoside Rd and bifidobacterial-fermented ethanol-extracted red ginseng could alleviate allergic rhinitis by suppressing IgE, IL-4, IL-5, and IL-13 expression and restoring the composition of gut microbiota [63]. Zhang et al. [64,65] reported that ginsenoside Rd significantly inhibited the production of pro-inflammatory cytokines and mediators in carrageenan-induced rat paw edema; the detailed mechanisms might be related to reducing the inflammatory cell infiltration into inflammatory sites, inhibiting the tissue lipid peroxidation and increasing the antioxidant enzyme activities through downregulation of NF-κB activation.

Additionally, oxidative stress-induced cell damage has been implicated in a variety of disease, such as aging, neurodegenerative disorders and certain chronic diseases. Ginsenoside Rd could be considered a potential antioxidant agent for prolonging the lifespan in senescence-accelerated mice and *C. elegans* [66,67]. Moreover, ginsenoside Rd was reported to have an anti-oxidative effect by enhancing glutathione levels in H4IIE cells via NF-κB-dependent γ-glutamylcysteine ligase induction [69]. Ye and co-workers investigated the protective role of ginsenoside Rd against the cytotoxicity in PC12 cell lines induced by exposure to hydrogen peroxide, indicating the potential neuroprotective effects [68].

### 3.4. Cognition and Neuroprotection

**Figure 4:**
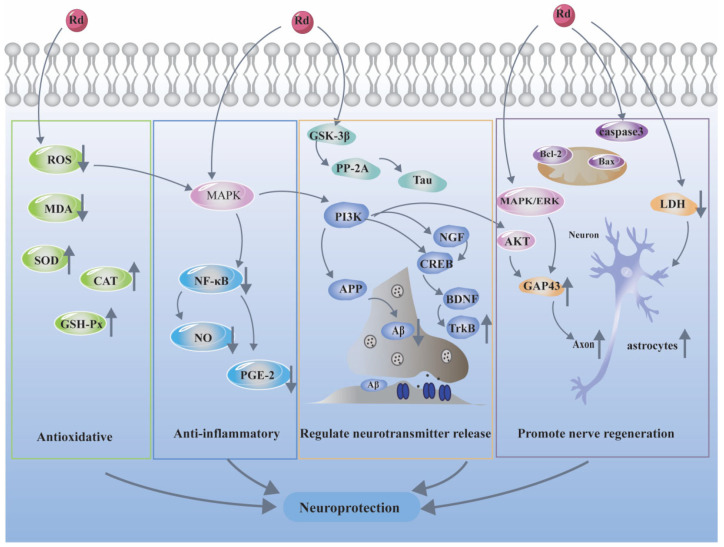
Neuroprotective mechanism of ginsenoside Rd. “↓” means downregulation, “↑” means upregulation.

Various ginseng species and ginsenosides have been documented to possess therapeutic effects in many central nervous system (CNS) ailments, for instance, Alzheimer’s disease, Parkinson’s disease, spinal cord injury, depression, and other cognitive impairment. The protective effects could be ascribed to reducing neuroinflammation, improving oxidative stress, regulating neurotransmitter release, and promoting nerve regeneration. Recent studies have shown that ginsenoside Rd could be a promising natural neuroprotective agent [4]. The current review summarizes the recent progress in neuroprotective effects of ginsenoside Rd in detail (Table 4, Figure 4).

Alzheimer’s disease (AD), is a neurodegenerative disease characterized by the sophisticated and unknown pathogenesis. Currently, the main popular hypotheses include neuronal dysfunction triggered by deposition of amyloid β (Aβ) proteins, neurofibrillary tangles triggered by hyperphosphorylation of tau protein, and cholinergic nerve degeneration [70]. Several studies examined the neuroprotective effects of Rd against neuronal insults in Aβ_25–35_ or Aβ_1–40_ induced AD rat models by ameliorating oxidative stress, alleviating the inflammation and reducing neuronal apoptosis [71,72]. Rd could also improve learning and memory ability in Aβ-protein precursor (APP) transgenic mice through inhibiting the transcription activity of NF-κB [73]. Furthermore, ginsenoside Rd attenuated Aβ-induced pathological tau phosphorylation by altering the functional balance of GSK-3β and PP-2A in Aβ-treated cortical neurons and in Aβ_1–40_ induced rat model and APP transgenic mice model [74]. A deficiency of the neurotransmitter acetylcholine (ACh) is also the major characteristic of Alzheimer’s disease. Results by Kim revealed that Re and Rd effectively induced the expression of cholinergic markers ChAT/VAChT genes and elevated ACh in Neuro-2a cells, as well as played an important role in neuronal differentiation and the nerve growth factor (NGF)-TrkA signaling pathway [75]. Ginsenoside Rd reduced OA-induced neurotoxicity and tau hyperphosphorylation in OA induced rat model (10 mg/kg) or in cultured cortical neurons (2.5 or 5 μM for 12 h) by enhancing the activities of protein phosphatase 2A (PP-2A) indicating that Rd might be a potential preventive drug candidate for AD and other tau pathology-related neuronal degenerative diseases [76]. Another recent research documented the protective effects of Rd against ovariectomy rat model. Rd enhanced learning and memory function of ovariectomy rats by increasing levels of sAPPα in the hippocampi, reducing extracellular Aβ and activating estrogen-like activity [77].

Recent findings highlighted the efficacy of ginsenoside Rd as neuroprotective compounds for Parkinson’s disease (PD) prevention and treatment through reducing oxidative stress, improving mitochondrial integrity and functions, and inhibiting apoptosis [78,79,80]. As a potential neuroprotective agent, ginsenoside Rd exhibited anti-neurotoxicity effect on various neurotoxic injury responses induced by Pb, trimethyltin (TMT), or kainic acid (KA) [81,82,83]. Cong et al. [84] evaluated the neuroprotective effects of ginsenoside Rd in a rat model of spinal cord injury (SCI), and the results demonstrated that Rd (25 and 50 mg/kg) significantly improved the locomotor function of rats after SCI through reversing the redox-state imbalance, inhibiting the inflammatory response and apoptosis in the spinal cord tissue. Another study investigated the protective effects of Rd on spinal cord mitochondrial dysfunction by regulating mitochondrial permeability transition pore formation and cytochrome c release [85]. 

Additionally, several recent studies focused on the effects of stress-related disorders of Rd and other protopanaxatriol-type ginsenosides. Sustained stress has been considered a risk factor for human ailments, including depression, anxiety, and cognitive dysfunction. Brain-derived neurotrophic factor (BDNF), a neurotrophin, is crucial to the survival, growth, and maintenance of neurons involved in emotional and cognitive function in brain. Results of Han et al. [86] showed that Rd mitigated anxiety/depression, colitis and gut dysbiosis by regulating NF-κB-mediated BDNF expression. Moreover, Rd improved cognitive impairment in chronic restraint stress mice by mitigating oxidative stress and inflammation, while upregulating the hippocampal BDNF-mediated cAMP-reflecting element binding (CREB) protein signaling pathway [87]. Moreover, Rd ameliorated impairment of learning and memory behaviors in chronic cerebral hypoperfusion (CCH) mice through regulation of BDNF by reestablishing the balance between Ac-H3 and HDAC2 [88]. The occurrence of metabolic and psychiatric disorders may be caused by higher levels of glucocorticoids. Ginsenoside Rd could inhibit adrenocorticotrophic hormone (ACTH)-induced corticosterone production through blockading the MC2R-cAMP/PKA/CREB pathway in adrenocortical cells, which might represent an important therapeutic option for the treatment of stress-related disorders [89]. Ginsenoside Rd also exerted neuroprotective effects after noise-induced auditory system damage through a mechanism involving the SIRT1/PGC-1α signaling pathway, which could be an attractive pharmacological target for the development of novel drugs for noise-induced hearing loss treatment [90].

The effect of ginsenoside Rd on inducing neural stem cells differentiation remains to be obscure. One study showed that ginsenoside Rd enhanced the proliferation but did not affect the differentiation of neural stem cells in adult rats and cultured neural stem cells [91], however, another study illustrated that Rd promoted the differentiation of neurospheres into astrocytes in a dose-dependent manner [92]. PC12 cells respond to nerve growth factor (NGF) and could be serve as a model for neuronal cells. Wu et al. [93] provided the first evidence that Rd promoted the neurite outgrowth of PC12 cells by upregulating GAP-43 expression via ERK- and ARK-dependent signaling pathways. Furthermore, Rd inhibited glutamate-induced Ca^2+^ entry in a concentration-dependent manner and prevented glutamate-induced apoptosis in rat cortical neurons, which provided potential evidence of Rd as a new neuroprotective drug for the prevention of neuronal apoptosis and death induced by cerebral ischemia [94].

**Table 4:** Neuroprotective effects and the molecular mechanisms of Rd.

Disease Type	Cell Lines/Animal	Effective Concentration/Dose	Effects	Mechanisms of Action	Refs.	Year
Alzheimer’s disease (AD)	Animals: Aβ_1__–40_ induced AD rat model	In vivo: Rd (10, 30 mg/kg/d, 30 days)	Protected cognitive impairment, improved memory function, alleviated Aβ_1–40_ induced inflammation	caspase-3↓, apoptosis↓	[71]	2012
	Cell lines: Aβ_25–35_ induced primary hippocampal neurons	In vitro: Rd (0.1, 1, 10 μM)	Ameliorated Aβ_25–35_ induced damage in primary cultured hippocampal neurons, inhibited Aβ_25–35_ induced apoptosis and oxidative stress, reversed Aβ _25–35_ induced alterations	ROS↓, MDA↓, GSH-Px↑, SOD↑, Bcl-2↑, Bax↓, Cyt c↓, c-caspase-3↓	[72]	2015
	Animals: APP transgenic mice	In vivo: Rd (10 mg/kg)	Improved learning and memory ability in APP transgenic mice	NF-kB↓	[73]	2015
	Cell lines: cortical neurons from mice E17–18 embryosAnimals: Aβ_1–40_ induced AD rat model and APP transgenic mice	In vitro: Rd (2.5, 5 μM, 12 h) In vivo: Rd (5 mg/kg)	Inhibited OA-induced tau phosphorylation in vivo and in vitro	Altered the functional balance of GSK-3β and PP-2A	[74]	2013
	Cell lines: Neuro-2a	In vitro: Rd (2.5 to 5 µg/mL)	Enhanced the expression of cholinergic markers and neuronal differentiation	ChAT/VAChT↑, ERK and AKT↓; MAP-2↑, p75↑, p21↑, NGF-induced TrkA↑	[75]	2014
	Animals: OA induced AD rat model	In vivo: Rd (2.5, 5 μM)	Protected SD rats and cultured cortical neurons against OA-induced toxicity	Decreased OA-induced the hyperphosphorylation of tau by the increase in activities of PP-2A	[76]	2011
	Animals: ovariectomy (OVX) rat model	In vivo: Rd (10 mg/kg, 2 months)	Enhanced learning and memory function of OVX rats and attenuated cognitive and memory impairment	α-Secretase and sAPPα↑, β-secretase and Aβ↓, p-ER-α at Ser118 residue↑	[77]	2017
Parkinson’s disease (PD)	Cell lines: SH-SY5Y	In vitro: Rd (0.5, 1 μM, 24 h)	Reduced oxidative stress, improved mitochondrial integrity and functions, and inhibited apoptosis	Bax/Bcl-2↓, Cyt c↓, caspase-3↓	[78]	2017
	Cell lines: SH-SY5Y	In vitro: Rd (1, 10 μM)	Exerted protective effect on neurodegenerative diseases, attenuated MPP^+^-induced cell death	Oxidative stress↓, mitochondrial function↑ and inhibited MPP^+^ induced ATP depletion, Bax/Bcl-2↓, Prevented p-AKT downregulation induced by MPP^+^ treatment	[79]	2015
	Cells: CCL_4_-treated primary dopaminergic cell cultures	In vitro: Rd (1, 5, 10 µM)	Protected dopaminergic neurons against CCl_4_-induced neurotoxicity; inhibited both oxidative stress and inflammation	LDH↓, NO↓, superoxide formation↓	[80]	2016
Neurotoxicity	Animals: lead (Pb)-treated old rat model	In vivo: Rd (50 mg/kg/d, 7 days)	Neuroprotective effects in old rats following acute Pb exposure	IL-1β↓, IL-6↓, TNF-α↓	[81]	2013
	Cells: TMT-treated hippocampal neurons	In vitro: Rd (1–40 µg/mL, 24 h)	Prevented TMT-induced cell apoptosis; attenuated the tremor seizures and cognitive decline; reduced neuronal loss	Bcl-2↑, Bcl-2↓, caspase-3↓	[82]	2017
	Animals: KA-induced ICR mice	In vivo: Rd (50 mg/kg)	Attenuated the KA-induced lethal toxicity	p-ERK↑ and p-CREB↓	[83]	2003
Spinal cord injury (SCI)	Animals: spinal cord injury (SCI) rat model	In vivo: Rd (12.5, 25, 50 mg/kg)	Attenuated SCI-induced secondary injury through reversing the redox-state imbalance, inhibiting the inflammatory response and apoptosis	MAPK↓, MDA↓, GSH and SOD↑, TNF-α, IL-1β↓	[84]	2016
	Mitochondria isolated from mouse spinal cord tissues Animals: male C57BL/6J mice	In vitro: Rd (0.1, 1, 10 µM, 60 s) In vivo: Rd(10, 50 mg/kg, 7 days)	Protected isolated spinal cord mitochondria against Ca^2+^ induced MPT and cytochrome c release in a mitochondrial protein kinases-dependent manner	Ca^2+^ induced Cyt c↓, intramitochondrial AKT and ERK↑	[85]	2014
Stress-related disorders	Animals: immobilization stress (IS) or *Escherichia coli* (*E. coli*)-treated anxiety/depression mice model	In vivo: Rd (5 mg/kg/d, oral, 5 days)	Alleviated the IS-induced anxiety/depression and *E. coli*-induced anxiety/depression, colitis, and gut dysbiosis in mice	Myeloperoxidase activity↓, NF-κB↓, NF-κB^+^/CD11c^+^ cell population↓	[86]	2020
	Animals: CRS induced cognitive impairment mice model	In vivo: Rd (20, 40 mg/kg/d, 28 days)	Improved cognitive impairment subjected to chronic stress	Oxidative stress↓, inflammation↓, hippocampal BDNF-mediated CREB signaling pathway↑	[87]	2020
	Animals: chronic cerebral hypoperfusion (CCH) mice model	In vivo: Rd (10, 30 mg/kg/d, 21 days)	Ameliorated CCH-induced impairment of learning and memory behaviors	Neuron survival↑, BDNF expression↑	[88]	2016
	Cell lines: mouse adrenocortical tumor cell line Y1	In vitro: Rd (2 μM)	Inhibited corticosterone secretion in the cells and impeded ACTH-induced corticosterone biosynthesis	cAMP/PKA/CREB signaling pathway↓; attenuated the induction of MC2R and MRAP by ACTH	[89]	2020
Noise-induced hearing loss (NIHL)	Animals: noise-induced guinea pigs	In vivo: Rd (30 mg/kg, i.p.)	Exerted neuroprotective effects after noise-induced auditory system damage; ameliorated auditory cortex injury associated with military aviation NIHL	SIRT1/PGC-1α signaling pathway↑	[90]	2020
Neural cells	Cells: neural stem cells Animals: male SD rats (180–220 g)	In vitro: Rd (0.1, 1, 10, 50 μM) In vivo: Rd (10, 30 mg/kg)	Had beneficial effects on learning and memory, promoted the size and number of neurospheres; but did not affect the differentiation of neural stem cells into neurons, astrocytes and oligodendrocytes	/	[91]	2012
	Cells: neural stem cells	In vitro: Rd (0.1, 1 μM)	Promoted the differentiation of neurospheres into astrocytes and increased the production of astrocytes	Number of neurons↓, astrocytes↑	[92]	2005
	Cell lines: PC12	In vitro: Rd (10 µM)	Promoted the neurite outgrowth of PC12 cells	GAP-43↑ via ERK and ARK signaling pathways	[93]	2016
	Cells: rat cortical neurons	In vitro: Rd (1, 3, 10, 30 µM)	Prevented glutamate-induced apoptosis in rat cortical neurons	Inhibited voltage-independent Ca^2+^ entry	[94]	2010

“/” means not mentioned, “↑”means upregulation, “↓”means downregulation.

### 3.5. Ischemic Stroke

In previous articles, the promising role of ginsenoside Rd on ischemic stroke has been described [3,95], the underlying mechanisms include the suppression of oxidative stress and inflammation, activation of PI3K/AKT pathway, suppression of the NF-κB as well as reduction of cytochrome c-releasing and apoptosis-inducing factor and so on. Thus, current review summarized the recent progress of Rd on ischemic stroke from 2015 to 2020 and focused on the molecular mechanisms underlying the beneficial role of ginsenoside Rd on ischemic stroke (Table 5).

There are multiple molecular mechanisms of ischemic stroke, of which oxidative DNA damage can trigger dysfunction and death of brain neurons and eventually lead to poor outcomes. The endonuclease VIII-like (NEIL) proteins NEIL1, NEIL2, and NEIL3, are major DNA glycosylases that remove oxidative base lesions. Yang et al. [96] investigated the effect of Rd on the expression of NEILs in the MCAO rat model and found that Rd significantly upregulated NEIL1 and NEIL3 expressions in both mRNA and protein levels.

The NMDA receptor (NMDAR) is a major excitatory neurotransmitter in central nervous system, which is involved in the pathological process of central nervous system diseases such as cerebral infarction, cerebral hemorrhage, ischemic stroke, brain trauma, etc. A recent study found that Rd could exert an inhibitory effect on NMDAR-triggered currents and sequential excitotoxicity through mitigation of DAPK1-mediated NR2B phosphorylation by attenuating calcineurin activity [97]. Rd protected ischemia–reperfusion injury (IRI) models rats and cultured neurons via inhibiting the hyperphosphorylation of NMDAR 2B subunit (NR2B subunit) and decreasing its expression levels in cell membrane [98].

It was well known that inflammation played an important role in the pathogenesis of ischemic stroke; however, the detailed mechanism of inflammatory modulation after ischemic stroke remained elusive. Microglia, the main immune cells in brain, are activated and subsequently release proinflammatory cytokines and other inflammatory mediators, worsening the neurologic outcome for stroke patients. Zhang et al. [99] demonstrated that Rd could safely improve the outcome of patients with ischemic stroke and revealed that administration of Rd in middle cerebral artery occlusion rat models could significantly inhibit ischemia-induced microglial activation and proteasome activity in microglia. Then, in 2020, the same research team further illuminated the downstream mechanisms that Rd was efficient for attenuating the pathogenesis of cerebral ischemia-induced blood–brain barrier damage by suppressing proteasome-mediated inflammation and sequentially suppressing NF-κB/MMP-9 pathway [100]. Moreover, nod-like receptor protein 3 (NLRP3) inflammasome plays a key role in mediating inflammatory response in the process of cardiovascular disorder, diabetes and ischemic stroke. It was reported that the combination of Panax ginseng and Angelica sinensis treatment attenuated cerebral injury via inhibition of NLRP3 inflammasomes activation and microglial pyroptosis after stroke, along with Drp1-mediated mitochondrial fission [101].

### 3.6. Cardiovascular Protection

A previous study has shown that ginsenoside Rd blocked Ca^2+^ influx through receptor- and store-operated Ca^2+^ channels in vascular smooth muscle cells, which might contribute to the cerebrovascular benefits [102] (Table 5). Nowadays, there is growing evidence that cerebrovascular remodeling is the common pathological basis of hypertension target organ damage, and circulatory dysfunction. Thus, effective cerebrovascular remodeling reversal therapy is an important measure to improve the prognosis of patients with hypertension, atherosclerosis, etc. Guan et al. [103] examined the effects of ginsenoside Rd on blood pressure, cerebrovascular remodeling and Ca^2+^ entry in freshly isolated basilar arterial vascular smooth muscle cells (BAVSMCs). Results showed that the attenuation of hypertensive cerebrovascular remodeling after Rd treatment, which the underlying mechanism might be associated with inhibition of voltage-independent Ca^2+^ entry and basilar artery smooth muscle cells (BASMCs) proliferation. Later, they investigated whether Rd influenced H_2_O_2_-induced apoptosis in BAVSMC [104]. The data strongly provided evidence that Rd potentiated H_2_O_2_-induced apoptosis of BASMCs through the mitochondria-dependent pathway. Then, ginsenoside Rd, as a voltage-independent Ca^2+^ channels blocker, reduced ox-LDL uptake and cholesterol accumulation in macrophages via inhibition of scavenger receptor A activity and expression, suggesting that Rd prevented the development of atherosclerosis [105]. Additionally, Lu et al. [106] focused on the effects of Rd on L-type calcium channel current in isolated rat ventricular myocytes and its potential mechanism and drew a conclusion that Rd might exert its protective effects via blocking of Ca^2+^ channel in cardiomyocytes. 

Cardiac hypertrophy, the gradual compensatory function of chronic stress load, eventually leads to myocardial ischemia and chronic heart failure. Results from Zhang et al. [107] revealed that ginsenoside Rd improved cardiac dysfunction and remodeling induced by pressure overload, which was related to the inhibition of protein levels of AKT, calcineurin A, ERK1/2 and TGF-β1. Myocardial ischemia–reperfusion (MI/R) injury refers to the structural and functional damage of myocardial cells caused by ischemic myocardium after resuming blood reperfusion; the mechanisms are still diverse and unknown. Rd-mediated cardioprotective effects against myocardial ischemia/reperfusion were found by both reducing intracellular reactive oxygen species, inhibiting mitochondria-mediated apoptosis and Ca^2+^ influx. Evidence suggested that Rd attenuated myocardial ischemia/reperfusion injury via inhibition of AKT/GSK-3β signaling and mitochondria-dependent apoptotic pathway in MI/R injury rat model and an in vitro neonatal rat cardiomyocyte (NRC) model [108]. Another report showed that Rd protected against MI/R injury as evidenced by improving cardiac function, decreasing infarct size and levels of serum creatine kinase, LDH and cTnI via Nrf2/HO-1 signaling, which played a key role in attenuating oxidative stress [109]. A recent study investigated the potential protective efficacy of Rd against nicotine-induced vascular endothelial cell injury by preserving normal vascular endothelial NO signaling, suppressing platelet aggregation and vasoconstriction, and by preventing endothelial cell-monocyte adhesion [110].

### 3.7. Immunological Activities

Recently, more attention has been paid to the effect of ginsenoside Rd on immune regulation in many immune-mediated diseases (Table 5). Multiple sclerosis (MS), one of the most common central nerve demyelinating diseases, is an autoimmune inflammatory disease affecting the central nervous system of the body. Ginsenoside Rd effectively ameliorated the clinical severity in EAE mice, providing a potential for amelioration of neuroimmune dysfunction diseases [111]. While the underlying mechanism of Rd in inhibiting the clinical course of EAE remains unclear. Furthermore, recent study investigated the potential mechanisms underlying the efficacy of Rd in alleviating the injury of EAE by modulating inflammation and autoimmunity via the downregulation of related proinflammatory cytokines IL-6 and IL-17, upregulation of inhibitory cytokines TGF-β and IL-10, and modulation of Treg/Th17 imbalance [112]. Similarly, Guillain–Barré syndrome (GBS) is also one of the most common immune-mediated neuropathies, characterized by demyelination and axonal damage, mainly peripheral nerve demyelination. A recent study by Ren et al. [113] provided the evidence of preventive effect of Rd on GBS by modulating monocyte subsets conversion and elevating the transcription factors, such as Nr4a1, through the in vivo experimental autoimmune neuritis mice model and in vitro mouse bone marrow stem cells.

Organ transplant rejection, a manifestation of the body’s immune response, seriously affects the prognosis of patients. It was reported that ginsenoside Rd could effectively antagonize transplant rejection via regulating the balance of Th1/Th2 type cytokines secretion, as well as reducing the percentages of CD4+ T cells and CD8+ T cells in the peripheral blood of rat recipients [114]. In parallel, ginsenoside Rd from Panax ginseng could enhance Th1 immunity, which might qualify Rd as an immunoadjuvant to induce surface mannan extract to produce a protective antibody [115,116]. However, it was noted that ginsenoside Rd and 20(S)-Rg3 isolated from red ginseng were identified as potential allergens that induced the release of mediators associated with anaphylactoid reactions [117].

### 3.8. Others

In addition, ginsenoside Rd was reported to have renal protection, lung protection, promotion of wound healing and bone differentiation, weight loss and other pharmacological activities (Table 6). Yokozawa and coworkers [118,119] evaluated the protecting effects of Rd against cisplatin-induced renal injury, a process in which apoptosis played a central role. Another study demonstrated that Rd possessed a protective function against renal ischemia/reperfusion injury (IRI) via downregulating M1 macrophage polarization [120]. Likewise, the protective effect of Rd on lipopolysaccharide (LPS)-induced acute lung injury (ALI) was recently investigated to explore the improvement of survival in endotoxemic mice by inhibiting the PI3K-AKT signaling pathway [121]. 

Ginsenoside Rd also had beneficial effects on weight loss, skin whitening and hair growth. Ginsenoside Rb1 and Rd were reported as representative compounds for improving the accelerated movement of the small intestine [122]. The beneficial effects of ginsenoside Rd on obesity and insulin resistance were found by Yao and co-workers in 2020, and its mechanisms were related to upregulation of thermogenesis in a cAMP-dependent manner [123]. Moderate melanogenesis inhibition activity of Rd at 20 μM purified from Panax ginseng berry in in melan-a cells, while floralginsenoside A (FGA) was observed to display the most potent inhibitory effect, which the potential whitening mechanism might be related to inhibition of melanin content and tyrosinase activity [124]. Moreover, ginsenoside Rb and Rd were reported to promote cell proliferation in HFs through p63 induction in follicular keratinocytes, which might be the therapeutic agent for the prevention of hair loss [125]. Kim et al. [126] identified ginsenoside Rd as the most active anti-osteoporotic agents via inducing the differentiation and mineralization of MC3T3-E1 cells through the activation of the AMPK/BMP-2/Smad signaling pathways. Moreover, ginsenoside Rd was screened through a Duchenne muscular dystrophy (DMD) hiPSC-derived myoblast screening platform and identified to significantly ameliorate some of the skeletal muscle phenotypes caused by dystrophin deficiency [127]. Later, the wound-healing effect of the ginsenoside Rd isolated from ginseng leaves was tested through in vitro the keratinocyte progenitor cells (KPCs), human dermal fibroblasts (HDFs) and animal wound models [128]. Ginsenoside Rd was also reported to prevent and rescue rat intestinal epithelial cells from irradiation-induced apoptosis [129]. 

## 4. Pharmacokinetics and Clinical Studies

Researchers have focused on ginsenoside Rd for its bioactivities; however, little is known about its pharmacokinetic behavior, solubility, bioavailability, safety, and clinical efficacies. A systematic and comprehensive understanding of Rd is not only necessary to further study its pharmacological actions and protective mechanisms, but to also provide a scientific basis for the clinical application of Rd and the development of new dosage forms. This review provides a profile of the pharmacokinetics, metabolism, safety, tolerance, and clinical efficacy of Rd (Table 7). 

### 4.1. Preclinical Studies

Previous literature reviewed the validity of Rd as a neuroprotective agent for acute ischemic stroke, including the pharmacokinetics, pharmacodynamics, clinical efficacy, safety, and putative therapeutic mechanisms of Rd [95]. In fact, in 2007, Wang et al. [130] first reported on the pharmacokinetic studies of ginsenoside Rd in dog plasma by liquid chromatography–mass spectrometry after solid-phase extraction. Sun et al. [131] analyzed the pharmacokinetic, tissue distribution, and excretion of ginsenoside Rd in rodents performed by HPLC and radioactive tracer assays. Results showed that intravascular administration with 20, 50, or 150 mg/kg Rd was rapidly distributed to various tissues; the dynamic changes were consistent with a two-compartment mode. Then, pharmacokinetic characteristics of eight ginsenosides, including Rd, Rh1, Rh2, Rg1, Rg2, Rg3, and so on, were investigated after an oral administration of GTSSL at a single dose of 400 mg/kg to rats based on the LC–ESI–MS/MS method [132]. Jeon et al. [133] investigated and compared ginsenoside pharmacokinetics in mice and rats following the repeated oral administration of red ginseng extract (RGE); results showed the pharmacokinetics and metabolic pathways of ginsenosides exhibited species differences. In mouse plasma, seven PPD-type ginsenosides (20(S)-Rb1, Rb2, Rc, Rd, Rg3, CK, and 20(S)-PPD) and one protopanaxatriol (PPT)-type 20(S)-Re were detected, whereas 20(S)-Rb1, Rb2, Rc, Rd, 20(S)-PPD, and 20(S)-PPT were detected in SD rat plasma. In addition, the T**_max_** and T**_1/2_** of 20(S)-PPD and 20(S)-PPT in rats were greater than those in mice, suggesting the species-dependent difference in the gut metabolism and absorption of ginsenosides. Recent study examined the pharmacokinetic profiles of ginsenosides Rd and Rg3 in mice orally gavaged with red ginseng (RG). Results showed that Rd absorbed was substantially high in fermented RG extract (fRGe)-treated mice, which suggested that oral administration of RG extracts could modify gut microbiome and consequently affect the bioavailability of RG ginsenosides [134]. Shenqi Jiangtang granule (SJG) is a traditional Chinese medicine prescription; Zhang et al. investigated the plasma pharmacokinetics during absorption of SJG after oral administration in rats [135]. The results showed that in vivo absorption and exposure of gomisin D and ginsenoside Rd were better than other analytes. It has been proven that some PPD-type ginsenosides, including G-Rb1, G-Rd, and partial PPT-type ginsenosides, have antidepressant and neuroregulatory effects. Recent literature reviewed the absorption and metabolism of Rd between normal and depressed rats [136]. As shown in Table 7, AUC values and Cmax values of Rd in the depression model group were increased and CL/F was decreased as compared with the normal group, suggesting the bioavailability of ginsenosides in the depression model could be improved.

### 4.2. Clinical Studies

In vivo pharmacokinetics and metabolism data of ginsenoside Rd may also be valuable for better understanding its pharmacologic activities and clinical application. In early 2007, Liu et al. Identified the metabolites of Rd in a rat and pharmacokinetic study in healthy volunteers [137]. Seven metabolites of Rd, mainly including oxygenation, glycosylation deglycosylation, were detected from rat urine collected from 0 to 24 h after oral and intravenous administration. The average half-life time of Rd in human plasma was detected as 19.29 h, indicating that the ginsenoside Rd may be metabolized slowly after intravenous administration. Later, the pharmacokinetics and safety of Rd were assessed in healthy Chinese participants through a phase I, randomized, open-label, single, and multiple-dose study [138]. Data showed that Rd was well tolerated with no dose-related adverse events, and had a good pharmacokinetics and safety profile, allowing it to be explored in future clinical studies in patients with acute ischemic stroke. Liu et al. [139,140] documented the improvement effects of Rd against acute ischemic stroke through a phase Π randomized, double-blind, placebo-controlled trial. In patients with acute ischemic stroke, Rd had favorable safety and tolerability, and improved the clinical symptoms of acute ischemic stroke. Two recent clinical trials showed that Rd had fewer side effects than glucocorticoid and could improve the outcome of patients with ischemic stroke by suppressing microglial proteasome activity and sequential inflammation [99].

Moreover, gut microbiota is not only involved in the biotransformation of ginsenosides, but also in the pharmacokinetics of ginsenosides in humans. However, few studies focused on the roles of the gut microbiota on the pharmacokinetics of ginsenosides in humans, and the effects had not yet been fully elucidated. A recent study determined the serum concentrations of the ginsenosides in humans after the administration of RG extracts (RG and fRG) and researchers analyzed their correlations with the fecal ginsenoside-metabolizing activities [141]. The gut bacteria seemed to exert their metabolic activity mainly on the biotransformation into ginsenoside CK via Rd rather than Rg3, suggesting that the profile and composition of the gut microbiota might affect the bioavailability and the pharmacological effects of ginsenosides. Overall, clinical studies on ginsenoside Rd are still limited, and mainly focused on phase 1 and phase 2 clinical studies of acute ischemic stroke, indicating that further studies on the potential efficacy of natural products in experimental animal models and randomized clinical trials are essential.

**Table 7:** Pharmacokinetics and clinical studies of Rd.

Compound	Subject	Dose	Pharmacokinetics Parameters						Ref.	Year
			C_max_ (ng/mL)	T_max_ (h)	AUC (ng h/L)	MRT (h)	CL/F (L·h^−1^)	T_1/2_ (h)		
**Preclinical Studies**										
Rd	Dogs	2 mg/kg, i.g.	81.0 ± 24.6	2.67 ± 1.17	1890.2 ± 668.6	25.5 ± 3.84	1.14 ± 0.40	24.2 ± 2.85	[130]	2007
		0.2 mg/kg, i.v.	/	/	76,403.4 ± 15,880.6	26.7 ± 1.63	0.0020 ± 0.0005	39.4 ± 12.0		
Rd	Kunming mice Wistar rats	20 mg/kg, 50 mg/kg, 150 mg/kg, i.v.	/	/	305.0 ± 22.3	/	0.066 ± 0.005	14.19 ± 2.37	[131]	2012
			/	/	293.2 ± 279.4	/	0.280 ± 0.172	12.83 ± 2.92		
			/	/	312.6 ± 139.5	/	0.569 ± 0.306	14.02 ± 10.57		
GTSSL	SD rats	400 mg/kg, i.g.	22.05 ± 2.21	2	2180.10 ± 18.69	12.43 ± 1.46	/	7.30 ± 3.32	[132]	2015
RG	ICR mice	2 g/kg/day, 7 days	51.7 ± 24.7	2.8 ± 3.3	1145 ± 555.6	/	/	40.1 ± 6.1	[133]	2020
	SD rats		6.5 ± 1.5	7.0 ± 2.0	257.8 ± 49.6	/	/	94.0 ± 23.7		
Rd	Wistar rats—normal	80 mg/kg, i.g.	97.458 ± 1.80	1.00 ± 0.01	2061.658 ± 1011.618	13.997 ± 0.390	64.895 ± 2.255	9.631 ± 0.206	[136]	2021
	Wistar rats—depression model		104.959 ± 5.0	1.00 ± 0.03	2583.439 ± 1254.680	15.126 ± 0.671	55.744 ± 2.366	10.198 ± 0.511		
**Clinical studies**										
Rd	199 + 390 patients	10, 20 mg, i.v.	/	/	/	/	/	/	[99]	2016
Rd	SD rats	60 mg/mL, i.v. 150 mg/kg, i.g.	/	/	/	/	/	/	[137]	2007
	10 healthy Chinese volunteers	10 mg, i.v.	2841.18 ± 473.03	0.50 ± 0.00	27261.63 ± 8116.88	17.52 ± 3.73	0.39 ± 0.12	19.29 ± 3.44		
Rd	24 healthy Chinese volunteers	10 mg	2.8 ± 0.5	0.5 ± 0.0	27.3 ± 8.1(mg·h/L)	17.5 ± 3.7	0.39 ± 0.12	19.3 ± 3.4	[138]	2010
		45 mg	10.5 ± 1.7	0.5 ± 0.0	112.6 ± 24.1 (mg·h/L)	18.3 ± 2.7	0.36 ± 0.08	18.4 ± 2.9		
		75 mg, i.v.	19.3 ± 2.6	0.5 ± 0.0	208.4 ± 51.4 (mg·h/L)	18.6 ± 2.7	0.37 ± 0.09	17.7 ± 2.0		
Rd	199 patients	10, 20 mg, i.v.	/	/	/	/	/	/	[139]	2009
Rd	390 patients	10 mg, i.v.	/	/	/	/	/	/	[140]	2012
RG	34 healthy Korean volunteers	3 g, i.g.	1.77 ± 2.09	15.12 ± 9.35	7.85 ± 11.24	/	/	/	[141]	2020

“/” means not mentioned.

## 5. Concluding Remarks

Collectively, this review systematically summarized recent advances on the biotransformation, pharmacological, pharmacokinetic, and clinical studies of ginsenoside Rd. Most pharmacological activities of Rd, including anti-cancer, anti-inflammatory, antioxidative, cardiovascular protection, and immunoregulation effects were exhibited and summarized. Other health-beneficial activities that have previously received less attention, such as kidney protection, lung protection, promotion of wound healing and bone differentiation, and anti-obesity, were also included. Moreover, the limited number of pharmacokinetic and clinical studies on ginsenoside Rd have also been documented.

Overall, Rd is a very promising candidate agent for the treatment of diverse diseases, while the following issues require greater attention in the future: (i) the exact mechanisms and targets that contribute toward the pharmacological activity of ginsenoside Rd require further detailed investigation; (ii) experimental animal model studies and randomized clinical trials should be performed to evaluate the therapeutic efficacy of ginsenoside Rd; (iii) the effects of ginsenoside Rd combined with chemotherapy, target therapy, or immunotherapy need to be determined.

## Figures and Tables

**Table 1:** Bioconversion of major ginsenosides into Rd.

Enzymes	Transformation Pathways	Optimum Conditions	Yield and Reaction Scale	Ref.	Year
Enzymatic Transformation					
**Arabinofuranosidase**					
α-l-arabinofuranosidase AbfA from Rhodanobacter ginsenosidimutans strain Gsoil 3054T	Rc ⤏ Rd	pH 7.5, 37 °C	/	[5]	2012
α-l-arabinofuranosidase, Abf22-3 from Leuconostoc sp. 22-3	Rc ⤏ Rd	pH 6.0, 30 °C	99.50%	[6]	2013
α-l-arabinofuranosidase from Caldicellulosiruptor saccharolyticus	Rc ⤏ Rd	pH 5.5, 80 °C, 227 U enzyme/mL	a molar yield of 100%	[7]	2013
α-l-arabinofuranosidase (Tt-Afs) from Thermotoga thermarum DSM5069	Rc ⤏ Rd	pH 5.0, 85 °C	99.40%	[8]	2016
α-l-arabinofuranosidase from Bacillus subtilis Str. 168	Rc ⤏ Rd	pH 5.0, 40 °C	90%	[9]	2021
**Arabinopyranosidase**					
α-l-Arabinopyranosidase from Blastococcus saxobsidens (AbpBs)	Rb2 ⤏ Rd	pH 7.0, 40 °C	/	[10]	2020
**β-glucosidase**					
β-glucosidase Tt-BGL from Thermotoga thermarum DSM 5069T	Rb1 ⤏ Rd	pH 4.8, 90 °C	95%	[11]	2013
β-glucosidase Bgp3 from Microbacterium esteraromaticum	Rb1 ⤏ Rd ⤏ CK	pH 7.0, 40 °C	77%	[12]	2012
glycosidase Bgp2 from Microbacterium esteraromaticum	Rb2 ⤏ Rd ⤏ 20(S)-Rg3	pH 7.0, 40 °C	65%	[13]	2013
β-Glucosidase Bgy2 from Lactobacillus brevis	Rb1 ⤏ Rd⤏F2 ⤏ CK	pH 7.0, 30 °C	69%91%	[14]	2016
β-glucosidase from Aspergillus niger KCCM 11239	Rb1 ⤏ Rd ⤏ Rg3 Rb1 ⤏ Rd ⤏ F2	pH 4.0, 70 °C	/	[15]	2012
**Pectinase**					
Pectinase coupled with one-pot process	Rb1 ⤏ Rd	pH 6.0, 52.5 °C	83.14%	[16]	2020
**Microbial Transformation**					
**Fungal System**					
*Paecilomyces bainier* 229-7	Rb1 ⤏ Rd	/	94.9% in shake flasks, 89% in 10 L fermenter	[17]	2010
*Paecilomyces bainier* 229-7	Rb1 ⤏ Rd	/	92.44%	[18]	2012
*Aspergillus versicolor* strain LFJ1403	Rb1 ⤏ Rd	pH 5.0, 37 °C	94.9% in shake flasks85% in 2 L fermenter	[19]	2015
*Aspergillus niger* strain TH-10a	Rb1 ⤏ Rd	pH 5.0, 32 °C	86%	[20]	2016
**Bacteria system**			/		
*M. trichothecenolyticum*	Rb1 ⤏ Rd⤏ Rh2	/	/	[21]	2013
Bacterial strain MAH-16T	Rb1 ⤏ Rd	pH 5.0–7.0, 20–40 °C	/	[22]	2018
Bacterial strain MAHUQ-46T	Rb1 ⤏ Rd	pH 7.5, 30 °C	/	[23]	2021
Bacterial strain FW-6T	Rb1 ⤏ Rd	/	/	[24]	2013
Bacterium G9y	Rc ⤏ Rd	pH 7.0, 45 °C	/	[25]	2021
**Gut microbiota**					
Gut bacteria	Rb1 ⤏Rd ⤏ F2 ⤏ CK Rb1 ⤏ G-XVII ⤏ G-LXXV ⤏ CK	/	/	[26]	2013
*Leuconostoc mesenteroides* DC102	Rb1 ⤏ G-XVII and Rd ⤏ F2⤏ CK	pH 6.0–8.0, 30 °C	99%	[27]	2011
*Lactobacillus paralimentarius* LH4	Rb1 ⤏ G-XVII and Rd ⤏ F2 ⤏ CK	pH 6.0, 30 °C	88%	[28]	2013
Probiotics	Rb1 ⤏ Rd ⤏ F2⤏ CK	/	/	[29]	2021
*Lactobacillus rhamnosus* GG	Rb1 ⤏ Rd	pH 6.0, 40 °C	/	[30]	2016
**Food microorganisms**					
*Dekkera anomala* YAE-1	Rb1 ⤏ Rd	pH 5.0, 40 °C	/	[31]	2020

“⤏” means convert to, “/” means not mentioned.

**Table 2:** Anti-cancer and anti-diabetic effects and the molecular mechanisms of Rb.

Anti-Cancer						
Disease Type	Cell Lines/Animal	Effective Concentration/Dose	Effects	Mechanisms of Action	Refs.	Year
Cervical cancer	Cell lines: HeLa	In vitro: IC_50_ = 150.5 ± 0.8 μg/mL (48 h)	Inhibited proliferation and induced cell apoptosis	Bcl-2↓, Bax↑, mitochondrial transmembrane potential↓, caspase-3↑	[34]	2006
Glioblastoma	Cell lines: U251	In vitro: IC_50_ = 88.89 μM (24 h); IC_50_ = 13.20 μM (28 h); IC_50_ = 9.55 μM (72 h)	Inhibited proliferation, promoted cell apoptosis, enhanced the expression of telomerase	caspase-3↑, Bcl-2↓, hTERT↓	[35]	2019
	Cell lines: U251, H4 (HTB148), U87 MG (HTB-14) cells, NHA	In vitro: Rd (100, 200 µM)	Reduced proliferation and migration	miR-144-5p↑	[36]	2020
Gastric cancer	Cell lines: SGC-7901Cell lines: MKN-45	In vitro: IC_50_ = 86.96 ± 0.23 μg/mL (SGC-7901, 48 h) and 71.70 ± 2.16 μg/mL (MKN-45, 48 h)	Inhibited proliferation, induced apoptosis and cell cycle arrest at G0/G1 phase	Cyclin D1↓, caspase-3↑, caspase-9↑, Bax/Bcl-2↑	[33]	2020
	Cell lines: AGS, MCF-7	In vitro: IC_50_ =131.2 μM (AGS) IC_50_ = 154.3 µM (MCF-7)	Inhibited proliferation	TRPM7 channel activity↓	[40]	2013
Liver cancer	Cell lines: HepG2	In vitro: EC_50_ = 18.26 μM	Combination of CA4P and Rd inhibited proliferation and induced apoptosis	HIF-1α↓, PI3K/AKT/mTOR↓	[38]	2021
	Cell lines: HepG2	In vitro: IC_50_ = 256.3 μM (24 h) and 172 μM (48 h)	Inhibited migration and invasion	MMP↓, MAPK↓	[41]	2012
Colorectal cancer	Cell lines: HT29	In vitro: IC_50_ = 277 μg/mL (48 h)	Inhibited proliferation	caspase 3↑, stathmin 1c, PCNA↓, rho GDP dissociation inhibitor (GDI) alpha↓, reticulocalbin 1 precursor↓, nudix hydrolase NUDT5↓, microtubule-associated protein RP/EB family↓, proteasome β 6 subunit↓, tyrosine 3/tryptophan 5-monooxygenase activation protein, epsilon↓, tropomyosin 1 (α)↑, glutathione S-transferase-P1↑, annexin 5↑, Nm23 protein↑, tropomodulin 3↑, and stratifin ↑	[37]	2009
	Cell lines: HT29 and SW620	In vitro: 0, 10, 50, 100 μM (72 h)	Inhibited metastasis	Bound to EGFR with a high binding affinity, stemness- and EMT-related genes↓	[42]	2019
	Cell lines: HUVEC animals: LoVo xenograft BALB/C mice	In vitro: Rd (2, 10, 50 µM In vivo: SMI (10 mL/kg/day, 13 days)	Suppressed neovascularization in tumors, normalized the structure of tumor vessels, and improved the anti-tumor effect of 5-FU	/	[45]	2019
	Animals: heterozygous C57BL/6J-Apc^Min/+^ mice	In vivo: Rd (20 mg/kg, 8 weeks)	suppressed cancer-promoting signaling markers, reduced the size and the number of the polyps, and improved intestinal barrier	iNOS↓, STAT3/pSTAT3↓, Src/pSrc↓, reinstated mucosal architecture, improved mucosal immunity, promoted beneficial bacteria, cancer cachexia associated bacteria↓	[48]	2017
Breast cancer	Cell lines: HEK293, MDA-MB-231, AU565, and T47D	In vitro: Rd (100–400 µM)	Suppressed the viability of TRPM7-expressing breast cancer cells	S phase↑, G0/G1 phase↓	[39]	2020
	Cell lines: AGS, MCF-7	In vitro: IC_50_ = 131.2 µM (AGS) and 154.3 µM (MCF-7)	Inhibited proliferation, induced cell apoptosis	TRPM7 channel activity↓	[40]	2013
	Cell lines: 4T1, MDA-MB-231	In vitro: Rd (50, 100, 150 μM, 72 h)	Suppressed cell migration and invasion	miR-18a-mediated Smad2↓	[41]	2016
	Cell lines: HUVECs, MDA-MB-231	In vitro: Rd (5, 10, 25, 50 µM)	Inhibited VEGF-induced migration, tube formation and proliferation of HUVECs, Inhibited proliferation and induced apoptosis	AKT/mTOR/P70S6↓	[42]	2017
	Cell lines: MCF-7, MCF-7/ADR	In vitro: Rd (10, 100 µg/mL, 24 h)	Reversed doxorubicin resistance in MCF-7/ADR cells	MDR1 protein↓	[44]	2010
Lung cancer	Cell lines: A549	In vitro: IC_50_ = 246.4 µM (24 h) IC_50_ = 149.0 µM (48 h) IC_50_ = 93.7 µM (72 h)	Inhibited proliferation, induced G0/G1 phase arrest, reversed cisplatin resistance	NRF2 pathway↓	[47]	2019
**Anti-diabetic**						
Diabetes	Animals: postnatal day 1 SD rats	In vivo: Rd (5, 10, 20, 50 μM)	Ameliorated the cell viability of MG-treated astrocytes	Improved insulin signaling and inhibited apoptosis	[49]	2014
	Cell lines: human pancreatic islets	In vitro: Rd (0.1,1,10 μM, 72 h)	Inhibited the progress of death of cultured human pancreatic islets, no effects on glucose-induced insulin and C-peptide stimulation secretion	Apoptosis of the islet cells↓, Bax↓, Bcl2↑, and caspase-3↓	[50]	2019
	Animals: type-2 diabetic db/db mice	In vivo: GS-E3D (100 or 250 mg/kg/d, oral, 6 weeks)	Renal protective roles	ROS↓	[51]	2021
Diabetic retinopathy (DR)	Cell lines: HUVEC Animals: STZ-induced diabetic mouse model	In vitro: Rd (1, 3, 10, 30 μM, 24 h) In vivo: Rd (100 mg/kg, 1 month)	Ameliorated diabetes-driven vascular damage, modulated oxidative stress and apoptosis	AMPK↑, SIRT1↑, AMPK/SIRT1 interaction↑	[52]	2022

“/” means not mentioned, “↑” means upregulation, “↓” means downregulation.

**Table 3:** Anti-inflammatory and antioxidative effects and the molecular mechanisms of Rd.

Anti-Inflammatory						
Disease Type	Cell Lines/Animal	Effective Concentration/Dose	Effects	Mechanisms of Action	Refs.	Year
Chronic hepatitis	Cell lines: HepG2	In vitro: Rd (IC_50_ = 12.05 ± 0.82 µM)	Anti-inflammatory activity	NF-kB↓, iNOS↓, COX-2↓	[53]	2012
Neuroinflammation	Cell lines: mouse primary neuron-glia Animals: pregnant OF1/SPF mice	In vivo: Rd (1, 10, 50 µM)	Protected dopaminergic neurons against LPS-neurotoxicity	iNOS↓, COX-2↓, iNOS↓, PGE2↓	[54]	2007
Osteoarthritis	Cell lines: S12	In vitro: Rd (100 μg/mL)	Exerted a protective effect against the cartilage degradation of OA	p-p38↓, MMP3↓	[55]	2009
Gastritis	Animals: ethanol- or indomethacin-induced gastric mucosal lesions in rat model	In vivo: Rd (100 mg/kg)	Showed gastroprotective effects on ethanol- and indomethacin-induced gastric mucosal lesions	/	[56]	2007
Colitis	Animals: DSS-induced murine colitis model	In vivo: Rd (10, 20, 40 mg/kg)	Ameliorated DSS-induced colitis, inhibited inflammatory cell recruitment into colonic tissue	p62-driven mitophagy-mediated NLRP3 inflammasome↓, AMPK/ULK1↑	[59]	2018
	Animals: TNBS-induced ulcerative colitis rat model	In vivo: Rd (10, 20, 40 mg/kg/d, orally	Against TNBS-induced recurrent ulcerative colitis and increased superoxide dismutase and glutathione peroxidase activities	Inhibited neutrophil infiltration and promoted the antioxidant capacity of the damaged colonic tissue	[60]	2012
	Animals: TNBS-induced ulcerative colitis rat model	In vivo: Rd (10, 20, 40 mg/kg/d, 7 days)	Attenuated the inflammatory response to TNBS-induced relapsing colitis	MPO↓, proinflammatory cytokine TNF-α, IL-1β, and IL-6↓, p-p38↓, JNK↓	[61]	2012
Inflammatory bowel diseases(IBD)	Animals: indomethacin-induced IBD rat model	In vivo: Rd (10, 20, 40 mg/kg, 7 days)	Stimulated the proliferation and differentiation of endogenous intestinal stem cells in IBD model rats, improved recovery of intestinal function	Bmi, CDX-2, and Msi-1↑	[62]	2020
Allergic rhinitis	Cell lines: RBL-2H3Animals: ovalbumin-induced AR mice model	In vivo: Rd (10 μM, 18 h)	Alleviated ovalbumin-induced allergic rhinitis in mice	IgE, IL-4, IL-5, and IL-13↓, restored the composition of gut microbiota	[63]	2019
Inflammatory	Cell lines: RAW264.7Animals: ICR mouse	In vitro: LPS (5 mg/kg) + Rd (2, 10, 50 mg/kg)	Anti-inflammatory effects	NF-kB↓, iNOS↓, COX-2↓, NO↓, PGE2↓	[57]	2013
	Cell lines: HepG2	In vitro: Rd (IC_50_ = 3.47 μM)	Suppressed inflammatory responses	NF-κB↓, COX-2↓ and iNOS↓	[58]	2014
	Animals: carrageenan-induced hind paw edema rat model	In vivo: Rd (12.5, 25, 50 mg/kg, i.m.)	Anti-inflammatory effects against carrageenan-induced edema	NF-kB↓	[64]	2012
	Animals: carrageenan -induced rat paw edema rat model	In vivo: Rd (12.5, 25, 50 mg/kg)	Reduced the inflammatory cell infiltration into inflammatory sites, inhibited the tissue lipid peroxidation, increased the antioxidant enzyme activities, and suppressed the proinflammatory enzyme expressions	NF-κB↓, p-ERK↓, p- JNK↓	[65]	2013
**Antioxidative**						
**Disease Type**	**Cell Lines/Animal**	**Effective Concentration/Dose**	**Effects**	**Mechanisms of Action**	**Refs.**	**Year**
Antioxidative	Animals: senescence-accelerated mice (SAM) of 10 months	In vivo: Rd (1 or 5 mg/kg/d, 30 days)	Attenuated the oxidative damage and enhanced the antioxidative defense system	Regulated the GSH/GSSG redox status	[66]	2004
	Animals: synchronized L4 larvae worms	In vivo: TG (10 μg/mL)	Has antiaging effects and only Rd prolonged the lifespan of C. elegans to levels comparable to total ginsenoside (TG)	Via lipid metabolism and activating the stress response signaling pathway	[67]	2021
	Cell lines: PC12	In vitro: Rd (1, 10 μM)	Antioxidative properties	ROS↓, MDA↓, SOD↑, GSH-Px↑, stabilized the mitochondrial membrane potential	[68]	2008
	Cell lines: H4IIE	In vitro: Rd (1–30 μg/mL)	Antioxidative effects; increased both cellular glutathione (GSH) content and the protein level of γ-glutamylcysteine ligase heavy chain	p65↑ via NF-κB-dependent γ-glutamylcysteine ligase induction	[69]	2007

“/” means not mentioned, “↑” means upregulation, “↓” means downregulation.

**Table 5:** Ischemic stroke, cardiovascular protection, and immunological activities and the molecular mechanisms of Rd.

Disease Type	Cell Lines/Animal	Effective Concentration/Dose	Effects	Mechanisms of Action	Refs.	Year
Ischemic stroke	Animals: MCAO rat models	In vivo: Rd (30 mg/kg)	Reduced mtDNA and nDNA damages and had the neuroprotective function	Survival rate and neurological function↑, cell apoptosis↓, cleaved caspase-3↓, NEIL1 and NEIL3↑	[96]	2016
	Cell lines: cortical neurons cells from embryonic day 18 SD ratsAnimals: MCAO rat models	In vitro: Rd (1, 3, 10, 30, 100 μM) In vivo: 10 mg/kg	Neuroprotectant for the treatment of ischemic stroke; exerted an inhibitive effect on NMDAR-triggered currents and sequential excitotoxicity	DAPK1-mediated NR2B phosphorylation↓, calcineurin activity↓	[97]	2020
	Cell lines: cortical neuronsAnimals: MCAO rat models	In vitro: Rd (10 μM) In vivo: Rd (50 mg/kg)	Improved the behavior score, infarct volume, and viability of the cultured neurons after ischemia	Hyperphosphorylation of NR2B subunit↓ and expression levels of NR2B subunit in cell membrane↓	[98]	2016
	Cells: microglia from P1 newborn SD rats, BV2, MC3T3-E1 Animals: MCAO rat models	In vitro: Rd (1, 10, 50, 100 μM) In vivo: 10 mg/kg, i.p.	Improved the outcome of patients with ischemic stroke	Microglial proteasome activity and sequential inflammation↓	[99]	2016
	Animals: MCAO rat models	In vivo: Rd (1, 10, 100 μM) In vivo: 30 mg/kg, i.p.	Attenuated the pathogenesis of cerebral ischemia-induced BBB damage, suppressed proteasome-mediated inflammation	Proteasome activity and NF-κB/MMP-9 pathway↓	[100]	2020
	Cell lines: BV-2 Animals: MCAO rat models	In vitro: Rd (0.1, 1, 10 μM) In vivo: CPA (4.5, 9 g/kg)	Attenuated cerebral injury after stroke	NLRP3↓, OGD/R-induced BV-2 cell injury↓, Drp1-mediated mitochondrial fission↓, Drp1↓	[101]	2020
Cardiovascular diseases	Cell lines: A10 embryonic rat thoracic aortic, rat aorta smooth muscle cells prepared from rat thoracic aorta	In vitro: Rd (100 μM)	Had an effect on cardiovascular diseases and inhibited Ca^2+^ entry	Through ROCC and SOCC without effects on VDCC and Ca^2+^ release	[102]	2006
Cerebrovascular remodeling	Cell lines: BAVSMCs from rat basilar arteries Animals: two-kidney, two-clip (2k2c) stroke-prone hypertensive rat model	In vitro: Rd(2.5, 5, 10, 20, 40 μM, 48 h) In vivo: Rd (20 mg in 2 mL saline solution containing 20% propylene glycol/kg/d)	Attenuated basilar hypertrophic inward remodeling in 2k2c hypertensive rats without affecting systemic blood pressure; attenuated hypertensive cerebrovascular remodeling	Inhibited voltage-independent Ca^2+^ entry and BAVSMC proliferation, but not with VDCC-mediated Ca^2+^ entry	[103]	2009
	Cell lines: BASMCs from rat basilar arteries	In vivo: Rd (10 μM)	Potentiated H_2_O_2_-induced cell death and cell apoptosis	Cyt c release↑, caspase-9/caspase-3↑, Bcl-2/Bax↓	[104]	2011
	Cell lines: RAW264.7 Animals: apolipoprotein E deficient (ApoE^−/−^) mice	In vitro, Rd (20 μM) In vivo: Rd (20 mg/kg/d)	Prevented the development of atherosclerosis	Through voltage-independent Ca^2+^ channels, SR-A↓, ox-LDL↓, cholesterol ↓	[105]	2011
	Cell lines: ventricular myocytes from the hearts of male SD rats	In vitro: Rd (IC_50_ = 32.4 ± 7.1 μM)	Protected the heart and inhibited ICa,L	ICa,L peak amplitude↓, the current-voltage (I-V) curve↑, changed the steady-state activation curve of ICa,L and slowed down the recovery of ICa,L from inactivation	[106]	2015
Cardiac hypertrophy	Cells: rat neonatal cardiac myocytes (NRCMs) from 24 h old SD ratsAnimal: C57BL/6 mice	In vitro: Rd (150 µg/mL) In vivo: Rd (50 μg/kg/d, i.v., 14 days)	Improved cardiac dysfunction and remodeling induced by pressure overload	AKT↓, calcineurin A↓, ERK1/2 and TGF-β1↓	[107]	2019
Myocardial I/R injury	Cells: neonatal rat cardiomyocytes (NRCs) Animals: MI/R injury rat model	In vitro: Rd (10 µM) In vivo: Rd (50 mg/kg)	Augmented rat cardiac function, reduced myocardial infarct size, apoptotic cell death	Left ventricular ejection fraction (LVEF)↑, ±dP/dt↑;inhibited caspase-9 and caspase-3, p-AKT and GSK-3β↑, and Bcl-2/Bax ratio↑	[108]	2013
	Cells: neonatal rat cardiomyocyte (NRCs) Animals: MI/R injury rat model	In vivo: Rd (50 mg/kg)	Improved cardiac function and attenuated myocardial infarction	Serum creatine kinase, LDH and cTnI↓, Nrf2, HO-1 and NQO1↑	[109]	2015
Vascular endothelial injury	Cell lines: HUVECs, THP-1Animal: nicotine-administered SD rat model	In vitro: Rd (30 μM, 24 h) In vivo: Rd (25, 50 mg/kg/d, 4 weeks)	Prevented nicotine-induced cardiovascular diseases	Vascular endothelial NO signaling↑, platelet aggregation and vasoconstriction↓, endothelial cell adhesion↓	[110]	2020
Multiple sclerosis (MS)	Animals: MOG_35–55_ induced EAE mouse model	In vivo: Rd (40 mg/kg/d, 35 days)	Ameliorated clinical severity and improved histopathology, reduced BBB dysfunction	IFN-γ↓, IL-4↑; BDNF and NGF↑	[111]	2014
	Cells: Mouse bone marrow stem cellsAnimals: EAE C57BL/6 mice	In vivo: 50 μM	Ameliorated the severity of EAE and attenuated the characteristic signs of disease; had modulation potential on gut microbiota in EAE mice	IL-6 and IL-17↓, TGF-β and IL-10↑, modulated Treg/Th17 imbalance	[112]	2020
Guillain–Barré syndrome (GBS)	Cells: mouse bone marrow stem cells Animals: P0180–199 induced EAN mouse model	In vitro: Rd(10, 30, 50 μM) In vivo: Rd (20, 50, 100 mg/kg, 30 days)	Preventive function on GBS, attenuated experimental autoimmune neuritis in mice	Modulated monocytes infiltration and macrophage polarization, regulated monocyte phenotype	[113]	2021
Immunosuppressive	Cells: mouse spleen T lymphocytesAnimals: allo-skin transplantation rat model	In vivo: Rd (25 mg/kg)	Antagonized transplant rejection	Th1 cytokines IL-2↓, IFN-γ↓, TNF-α↓, IL-12↓, Th2 cytokine IL-10↑	[114]	2012
Immunoadjuvant	Animals: OVA-immunized mouse model	In vivo: Rd (25 μg, 2 weeks)	Had immunological adjuvant activity, and elicited a Th1 and Th2 immune response, enhanced the Con A-, LPS-, and OVA-induced splenocyte proliferation	Regulated production and gene expression of Th1 cytokines and Th2 cytokines	[115]	2007
	Strains: *C. albicans* strains Animals: vaccinated BALB/c mice	In vitro: Rd (1 mg/mL) In vivo: Rd (1 mg/mL, i.p., 10 days)	Protected mice against disseminated candidiasis and enhanced Th1 immunity	Elicited higher titers of Th1 type antibody and a Th1-dominant immune response	[116]	2013
Anaphylactoid reactions	Cells: RBL-2H3 MCs, mouse peritoneal mast cells (MPMC) isolated from mouse, LAD2 cells Animals: ICR male mice (18–22 g)	In vitro: Rd (0.11, 0.21, 0.42 mM) In vivo: Rd (10, 20, 40 mg/kg)	Potential allergens, induced the release of mediators associated with anaphylactoid reactions	β-hexosaminidase↑, histamine↑, translocation of phosphatidylserine↑, Ca^2+^↑	[117]	2017

“↑”means upregulation, “↓”means downregulation.

**Table 6:** Other health-beneficial activities and the molecular mechanisms of Rd.

Disease Type	Cell Lines/Animal	Effective Concentration/Dose	Effects	Mechanisms of Action	Refs.	Year
Renal injury	Animals: cisplatin-induced acute renal failure rat model	In vivo: Rd (1, 5 mg/kg/d, 30 days)	Decreased the severity of renal injury induced by cisplatin	MDA↓, blood urea nitrogen↓, Cr↓, urinary excretion of glucose↓	[118]	2000
	Cell lines: LLC-PK1 cells cultured with cisplatin Animals: cisplatin-induced acute renal failure rat model	In vitro: Rd (125 μM) In vivo: Rd (1, 5 mg/kg/d, 30 days)	Ameliorated cisplatin-induced renal injury, caused restoration of the renal function	DNA fragmentation↓, apoptosis↓, urea nitrogen and creatinine↓	[119]	2001
	Cell lines: mouse polarized macrophagesAnimals: renal IRI mouse model	In vitro: Rd (10, 20, 50, 100 μg/mL) In vivo: Rd (10, 20, 50, 100 mg/kg)	Alleviated mouse acute renal ischemia/reperfusion injury	M1 macrophage polarization↓	[120]	2016
Acute lung injury (ALI)	Animals: LPS-induced ALI mouse model	In vivo: Rd (25, 50 mg/kg)	Protected mice against LPS-induced ALI; improved survival in endotoxemic mice	PI3K/AKT↓	[121]	2021
Small intestinal transport	Animals: carbachol/BaCl_2_-induced accelerated small intestinal transit mouse model	In vivo: Rd (0.4, 1.0, 2.0 mg/kg)	Ameliorative effects on the carbachol-induced accelerated small intestinal transport	Intestinal motility↓, cholinergic nervous system↓	[122]	2003
Anti-obesity	Animal: high-fat diet-induced obese mouse model	In vivo: Rd (15 mg/kg/d, 23 days)	Ameliorated obesity and insulin resistance	Cyclic adenosine monophosphate (cAMP)↑	[123]	2020
Whitening activity	Cell lines: Melan-a cellsAnimal: zebrafish	In vitro: Rd (10, 20 μM) Re (20, 40, 80 μM) FGA (20, 40, 80, 160 μM) In vivo: FGA (80, 160 μM)	Inhibited melanin biosynthesis	AKT↑, ERK↑	[124]	2017
Anti-alopecia	Cells: HFsAnimals: shaved skin B57CL/6 mouse model	In vivo: Rd and Rb1 (300 mg/kg/d, 35 days)	Promoted hair growth	p63 expression↑ in hair follicles	[125]	2012
Anti-osteoporotic	Cell lines: MC3T3-E1	In vitro: Rd (10, 20, 40 μM)	Stimulated osteoblastic differentiation and mineralization	AMPK/BMP-2/Smad signaling pathways↑	[126]	2012
Duchenne muscular dystrophy (DMD)	Cells: D2325 fibroblasts from a DMD patient Animals: mdx^5cv^ mice	In vitro: Rd (5 μM) In vivo: Rd (10 mg/kg)	Ameliorated some of the skeletal muscle phenotypes caused by dystrophin deficiency	FLT3 signaling↑	[127]	2020
Wound healing effects	Cell lines: KPCs, HDFsAnimal: hairless wound mice model	In vitro: Rd (0.1, 1, 10 μM) In vivo: Rd (10 μM, every 2 days, 10 days)	Promoted skin regeneration	Collagen type 1↑, matrix metalloproteinase-1 (MMP-1) ↓, cAMP-dependent protein kinase pathway↑	[128]	2013
Irradiation-induced damage	Cell lines: rat intestinal epithelial IEC-6 cells	In vitro: Rd (2.5, 5, 10, 20, 40 μM, 24 h)	Protected and rescued rat intestinal epithelial cells from irradiation-induced apoptosis	Bax/Bcl-xL↓, Cyt c↓, cleaved-caspase-3↓, PI3K/AKT↑, MEK↓, mitochondria/caspase pathway↓	[129]	2008

“↑”means upregulation, “↓”means downregulation.

## Data Availability

Not applicable.

## Abbreviations

2k2c	two-kidney, two-clip;
5-FU	5-fluorouracil;
ACh	acetylcholine;
Ac-H3	acetylated histone H3;
ACTH	adrenocorticotrophic hormone;
AD	Alzheimer’s disease;
ADR	Adriamycin;
ALI	acute lung injury;
AMP	adenosine monophosphate;
AMPK	adenosine 5‘-monophosphate (AMP)-activated protein kinase;
APP	Aβ-protein precursor;
Aβ	amyloid β;
BASMCs	basilar artery smooth muscle cells;
BAVSMCs	basilar arterial vascular smooth muscle cells;
BBB	blood–brain barrier;
Bax	Bcl2-Associated X;
Bcl-2	B-cell lymphoma-2;
BDNF	brain-derived neurotrophic factor;
Bmi-1	B cell-specific MLV insertion site-1;
C/EBP	CCAAT/enhancer binding protein;
CA4P	combretastatin A4 phosphate;
CaM	calmodulin;
CCH	chronic cerebral hypoperfusion;
CDX-2	caudal type homeobox 2;
ChAT	choline O-Acetyltransferase;
COX	cyclooxygenase;
CREB	cAMP-response element binding protein;
CRS	chronic restraint stress;
CPA	combination of Panax ginseng and Angelica sinensis;
cTnI	cardiac troponin I;
DAPK1	death associated protein kinase 1;
Drp1	dynamin-related protein 1;
DSS	dextran sulfate sodium;
EAE	experimental autoimmune encephalomyelitis;
EAN	experimental autoimmune neuritis;
ERK	extracellular regulated protein kinases;
EGFR	epidermal growth factor receptor;
EMT	epithelial–mesenchymal transition;
*E. coli*	*Escherichia coli*;
FGA	floralginsenoside A;
fRG	fermented red ginseng;
GAP-43	growth associated protein-43;
GBS	Guillain–Barré syndrome;
GS-E3D	pectin-lyase-modified ginseng;
GSH	glutathione;
GSSG	oxidized glutathione;
GSH-Px	glutathione peroxidase;
HDAC2	histone deacetylase 2;
HO-1	heme oxygenase-1;
HPLC	high performance liquid chromatography;
hiPSCs	human induced pluripotent stem cells;
hTERT	human telomerase reverse transcriptase;
HUVECs	human umbilical vascular endothelial cells;
HFs	hair follicles;
IBD	inflammatory bowel disease;
iNOS	inducible nitric-oxide synthase;
IRI	ischemia-reperfusion injury;
i.p.	intraperitoneal;
i.v.	intravenous;
i.g.	intragastrically;
IS	immobilization stress;
JNK	c-Jun N-terminal kinase;
KPCs	keratinocyte progenitor cells;
KA	kainic acid;
Cyt c	cytochrome c;
DMD	Duchenne muscular dystrophy;
LPS	lipopolysaccharide;
LVEF	left ventricular ejection fraction;
MDR1	multidrug resistance protein 1;
MI/R	myocardial ischemia- reperfusion;
MMP	mitochondrial membrane potential;
MEK	methyl ethyl ketone;
MS	multiple sclerosis;
Msi-1	Musashi-1;
MPO	myeloperoxidase;
MPP+	1-methyl-4-phenylpyridinium;
MPMC	mouse peritoneal mast cells;
MC2R	melanocortin 2 receptor;
MCAO	middle cerebral artery occlusion;
MDA	malondialdehyde;
mTOR	mammalian target of rapamycin;
NHA	normal human astrocytes;
NEIL	Nei-like DNA glycosylase;
NF-κB	nuclear factor kappa-B;
NGF	nerve growth factor;
NLRP3	nod-like receptor protein 3;
NMDA	N-methyl-D-aspartic acid;
TMT	trimethyltin;
Tt-Afs	thermostable α-l-arabinofuranosidase;
TRPM7	transient receptor potential melastatin 7;
NMDAR	N-methyl-D-aspartic acid receptor;
Nr4a1	nuclear receptor subfamily 4 group A member 1;
NRCMs	neonatal rat cardiac myocytes;
NRF2	nuclear factor erythroid 2-related factor 2;
NIHL	noise-induced hearing loss;
NSC	neural stem cell;
NSCLC	non-small-cell lung cancer;
OA	okadaic acid;
ox-LDL	oxidized low density lipoprotein;
PD	Parkinson’s disease;
LDH	lactate dehydrogenase;
LDL	low-density lipoprotein;
PGE2	prostaglandin E2;
LC-ESI-MS/MS	liquid chromatography–electrospray ionization tandem mass spectrometry
PPD	protopanaxadiol;
Rd	ginsenoside Rd;
ROS	reactive oxygen species;
ROCC	receptor-operated Ca2+ channels;
sAPPα	soluble amyloid precursor protein alpha;
SCI	spinal cord injury;
SD rats	Sprague–Dawley rats;
SIRT1	sirtuin 1 SAM, senescence-accelerated mice;
SR-A	scavenger receptor A;
STAT3	signal transducer and activator of transcription 3;
SMI	Shenmai injection;
PKA	protein kinase A;
SFI	Shenfu injection;
SOCC	store-operated Ca^2+^ channels;
TUNEL	TdT-mediated dUTP nick-end labeling;
TG	total ginsenoside;
TNBS	2,4,6-trinitrobenzenesulfonic acid;
TLC	thin-layer chromatography;
TNBS	2,4,6-trinitrobenzenesulfonic acid;
Treg	regulatory T cells;
TRPM7	transient receptor potential melastatin 7;
PP-2A	protein phosphatase 2A;
SOD	superoxide dismutase;
OVX	mice, ovariectomy mice;
PGC-1α	peroxisome proliferator-activated receptor-γ coactivator-1α;
TGF-β	transforming growth factor-β;
Th17	T helper cell 17;
Tt-Afs	thermostable α-l-arabinofuranosidase;
TNF-α	tumor necrosis factor-α;
ULK1	unc-51-like autophagy activating kinase 1;
VAChT	vesicular acetylcholine transporter;
VDCC	voltage dependent Ca^2+^ channel;
GTSSL	total saponins in the stems-leaves of Panax ginseng C. A. Meyer.
